# Early implementation of a perioperative nutrition support pathway for patients undergoing esophagectomy for esophageal cancer

**DOI:** 10.1002/cam4.4360

**Published:** 2021-12-21

**Authors:** Rebecca A. Carr, Caitlin Harrington, Christina Stella, Diana Glauner, Erin Kenny, Lianne M. Russo, Meghan J. Garrity, Manjit S. Bains, Smita Sihag, David R. Jones, Daniela Molena

**Affiliations:** ^1^ Thoracic Service Department of Surgery Memorial Sloan Kettering Cancer Center New York New York USA; ^2^ Department of Food and Nutrition Services Memorial Sloan Kettering Cancer Center New York New York USA

**Keywords:** cancer‐associated malnutrition, esophageal cancer, esophagectomy, standardized nutritional pathway, undernutrition

## Abstract

**Background:**

Unintentional weight loss and malnutrition are associated with poorer prognosis in patients with cancer. Risk of cancer‐associated malnutrition is highest among patients with esophageal cancer (EC) and has been repeatedly shown to be an independent risk factor for worse survival in these patients. Implementation of nutrition protocols may reduce postoperative weight loss and enhance recovery in these patients.

**Methods:**

We retrospectively identified all patients who underwent Ivor Lewis esophagectomy for EC from January 2015 to August 2019 from a prospectively collected institutional database. Patients who underwent surgery after the implementation of this protocol (September 2017–August 2019) were compared with patients who underwent resection before protocol implementation (January 2015–July 2017). Patients undergoing surgery during the month of protocol initiation were excluded.

**Results:**

Of the 404 patients included in our study, 217 were in the preprotocol group, and 187 were in the postprotocol group. Compared with the preprotocol group, there were significant reductions in length of hospital stay (*p* < 0.001), time to diet initiation (*p* < 0.001), time to feeding tube removal (*p* = 0.012), and postoperative weight loss (*p* = 0.002) in the postprotocol group. There was no significant difference in the incidence of postoperative complications, 30‐day readmission, or mortality rates between groups.

**Conclusions:**

Results of the present study suggest a standardized perioperative nutrition protocol may prevent unintentional weight loss and improve postoperative outcomes in patients with EC undergoing resection.

## Graphical abstract

Risk of cancer‐associated malnutrition is high among patients undergoing surgery for esophageal cancer. A standardized perioperative nutrition protocol may prevent unintentional weight loss and improve postoperative outcomes in these patients.

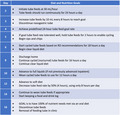

## 1 INTRODUCTION

Studies have repeatedly shown that unintentional weight loss and malnutrition in patients with cancer is associated with reductions in quality of life, treatment tolerance, and survival.^1^, ^2^ The prevalence of cancer‐associated malnutrition varies by tumor type but is commonly reported to be the highest in esophageal cancer (EC), affecting 60%–85% of patients at the time of diagnosis.^2^, ^3^, ^4^, ^5^, ^6^, ^7^, ^8^, ^9^ Preoperative weight loss in EC is most often the result of malignancy‐induced cachexia, mechanical obstruction of the esophagus causing dysphagia, and side effects of neoadjuvant treatment, such as nausea, vomiting, and anorexia.^1^, ^7^, ^10^, ^11^, ^12^ Postoperatively, difficulties maintaining adequate nutrition are often worsened as a result of permanent anatomical alterations to the gastrointestinal tract following esophagectomy resulting early satiety, postprandial dumping, frequent diarrhea, and regurgitation.^13^, ^14^, ^15^

Data indicate that dietitian‐delivered intensive nutritional support can improve postoperative weight loss and reduce the incidence of severe complications in patients undergoing esophagectomy.^16^, ^17^ However, there is no accepted standard of care to guide the optimal nutritional approach. To improve nutritional support for patients with EC undergoing esophagectomy at our institution, a perioperative nutritional care plan was developed with input from the Thoracic Surgery, Nursing, Food and Nutrition Service, and Clinical Nutrition Departments. Ultimately, a standardized protocol for perioperative nutrition management was established and subsequently implemented in August 2017 for all esophagectomy patients. The primary objective of the present study was to evaluate the impact of this program on length of stay, days to diet initiation, postoperative weight loss, and perioperative morbidity and mortality.

## 2 METHODS

Following institutional review board approval, we retrospectively queried our prospectively maintained database to identify all patients who underwent Ivor Lewis esophagectomy for histologically confirmed esophageal adenocarcinoma (EAC) or squamous cell carcinoma (ESCC) at our institution between January 2015 and August 2019. All relevant clinical and pathologic variables including baseline demographic characteristics, preoperative staging, tumor histologic characteristics and location, specific treatment regimens, and postoperative disease status were extracted from this database. Staging was performed using the 8^th^ edition of the American Joint Committee on Cancer (AJCC) Staging Manual of the tumor‐node‐metastasis classification.^18^

Two groups of patients were included in the present study. The postprotocol group (protocol+) included all patients with EC who underwent surgery after implementation of this standard perioperative protocol from September 2017 to August 2019. The preprotocol group (protocol−) included all patients who had undergone surgery between January 2015 and July 2017 before the implementation of the protocol. Patients who underwent surgery during protocol initiation (i.e. August 2017) were excluded. Other exclusion criteria included patients undergoing resection for benign lesions, other primary esophageal tumors other than ESCC or EAC, or tumors metastatic to the esophagus.

Height and weight were measured at three time points for each patient: at the initial outpatient visit before any treatment, immediately preoperatively, and postoperatively approximately 2 weeks from discharge at the first outpatient follow‐up visit. Body mass index (BMI) was calculated from height and weight and classified according to the National Institutes of Health (NIH) and World Health Organization (WHO) guidelines as either underweight (<18.5 kg/m^2^), normal weight (≥18.5–24.9 kg/m^2^), overweight (≥25.0–29.9 kg/m^2^), or obese (≥30 kg/m^2^).^19^, ^20^ Preoperative weight loss was reported as a percentage of the pretreatment weight and was calculated as the difference in weight from the pretreatment visit to surgery. Postoperative weight loss was reported as a percentage of the preoperative weight and was calculated as the difference in weight from surgery to first outpatient follow‐up visit. Significant weight loss was defined as a weight loss equal to or >10%.

### 2.1 Preprotocol group (protocol−)

Before our perioperative nutrition program was initiated, surgical attendings managed their patients individually without a standard of practice for nutrition in the postoperative setting. Surgical feeding tubes were placed at the attending's discretion. For patients without a feeding tube, intravenous fluid hydration was utilized until an oral diet began. For those with feeding tubes, the attending decided how and when to initiate and advance tube feeds and determined caloric needs.

### 2.2 Postprotocol group (protocol+)

**FIGURE 1:**
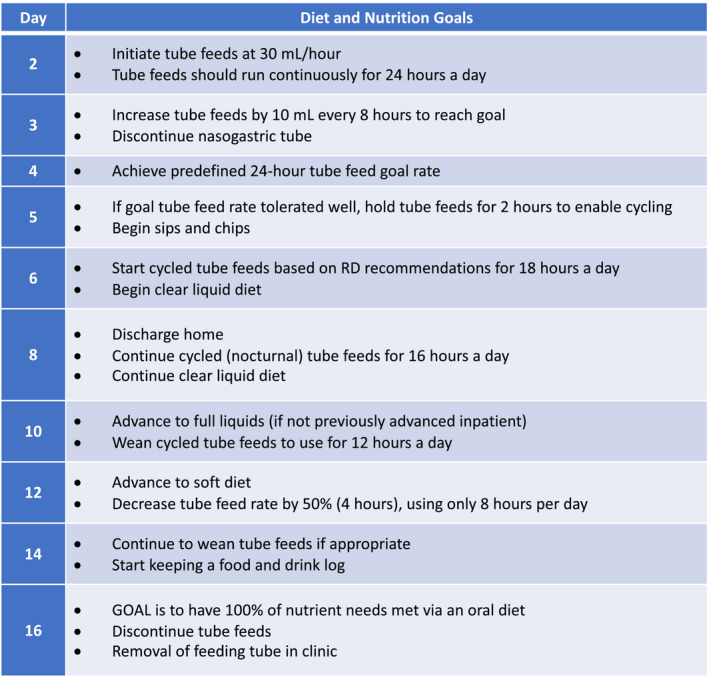
Standard perioperative nutrition protocol for all patients undergoing esophagectomy for esophageal cancer

**FIGURE 2:**
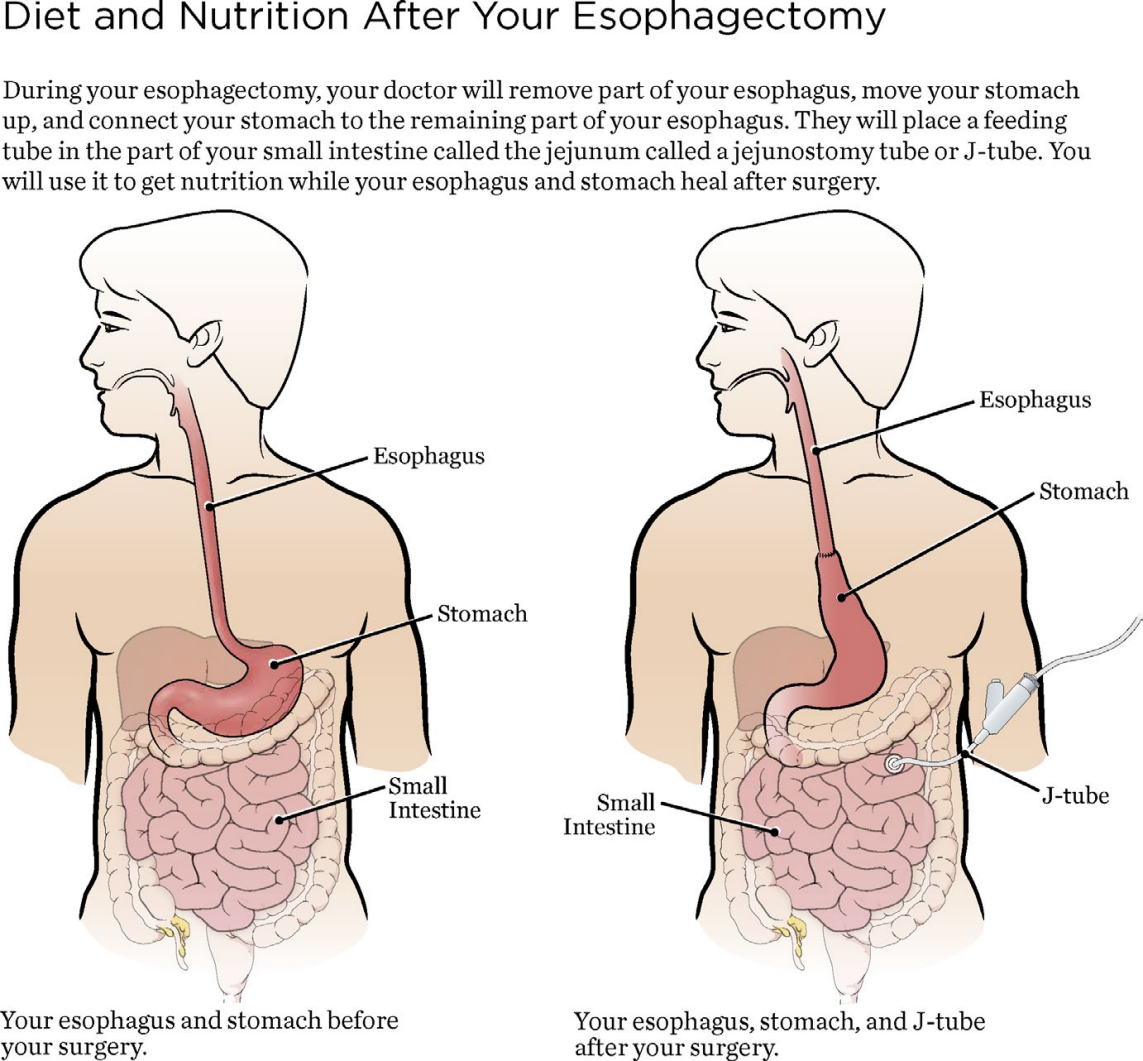
Patient information given as part of the nutrition perioperative program within the enhanced recovery program after esophagectomy

Our perioperative nutrition program consists of preoperative, intraoperative, and postoperative phases of management. Preoperatively, patients with EC are identified by a member of the surgical team following the informed consent process. Subsequently, an electronic nutritional consult is ordered via our institution's electronic medical record, which generates an email delivered directly to the dietitian's online mailbox. Patients are contacted within 48 h of the referral being received by the outpatient dietitian, who then reviews the patients’ presurgical nutritional status and provides nutrition counseling as needed. This appointment is crucial to screen for patients at increased risk of poor nutritional status, weight loss, and malnutrition, enabling providers to implement appropriate nutritional intervention preoperatively, such as addition of a nutrient‐enriched, high‐protein diet. Additionally, patients are given written handouts describing what to expect, as well as an activity and recovery log consisting of daily checklists that outline specific postoperative goals for each day (Figures 1 and 2).

Once the patient is admitted for esophagectomy, the inpatient dietitian assumes responsibility for nutrition care. In the postoperative phase, the inpatient dietitian evaluates the patient and calculates their goal caloric needs, typically ranging from 25 to 35 kcal/kg, with overweight patients receiving the lower end of that range. Tube feeding is initiated on postoperative day (POD) 2 via a surgical jejunostomy tube at 30 ml/h for 24 h with a standard polymeric formula providing 1.5 kcal/ml. Tube feeds are then titrated up to goal caloric value by POD 3 or 4. On POD 5, tube feeds are cycled to provide the equivalent calories over an 18‐h period at a maximum rate of 80 ml/h to promote tolerance, and clear liquid sips are initiated orally. Patients who are discharged on a clear liquid diet have their tube feeding goal reduced to 75% of their estimated needs over a 16‐h period to promote increased oral intake. Once the patient is deemed eligible for discharge, the inpatient dietitian notifies the outpatient dietitian, enabling continuity of care. The outpatient dietitian contacts the patient within 48 h of discharge and monitors the patient every 2–3 days by phone. These sessions allow for the dietitian to assess tolerance to the patient's enteral nutrition and liquid diet (clear vs. full liquid) and helps guide later diet advancement (e.g. advancing to full liquids if discharged on clears). Tube feeds are slowly weaned beginning on POD 12, when the patient is tolerating an oral diet, with the goal to initiate a soft diet with complete cessation of tube feeds by the 2‐week surgery follow‐up appointment.

### 2.3 Statistical analysis

Independent variables collected included 30‐day readmission, 30‐day mortality, 90‐day mortality, in‐hospital mortality, and incidence of serious postoperative complication, defined as any complication grade III or greater utilizing the Clavien–Dindo severity grading system.^21^ Categorical variables were summarized as frequencies and percentages and compared using the Fisher's exact and Chi‐Square tests as appropriate. Continuous outcome variables collected included postoperative weight loss, time in days from surgery to enteric tube removal, diet initiation, and feeding tube removal, and well as length of hospital stay. Continuous variables were summarized as medians and interquartile ranges and compared using the Kruskal–Wallis test for nonparametric data. Multivariable linear regression modeling was used to evaluate the impact of our nutrition protocol on all continuous outcome variables. All *p* values reported were two‐tailed, and statistical significance was defined as a two‐sided *p* < 0.05. Data were analyzed using Stata (Stata‐Corp, version 16).

## 3 RESULTS

**FIGURE 3:**
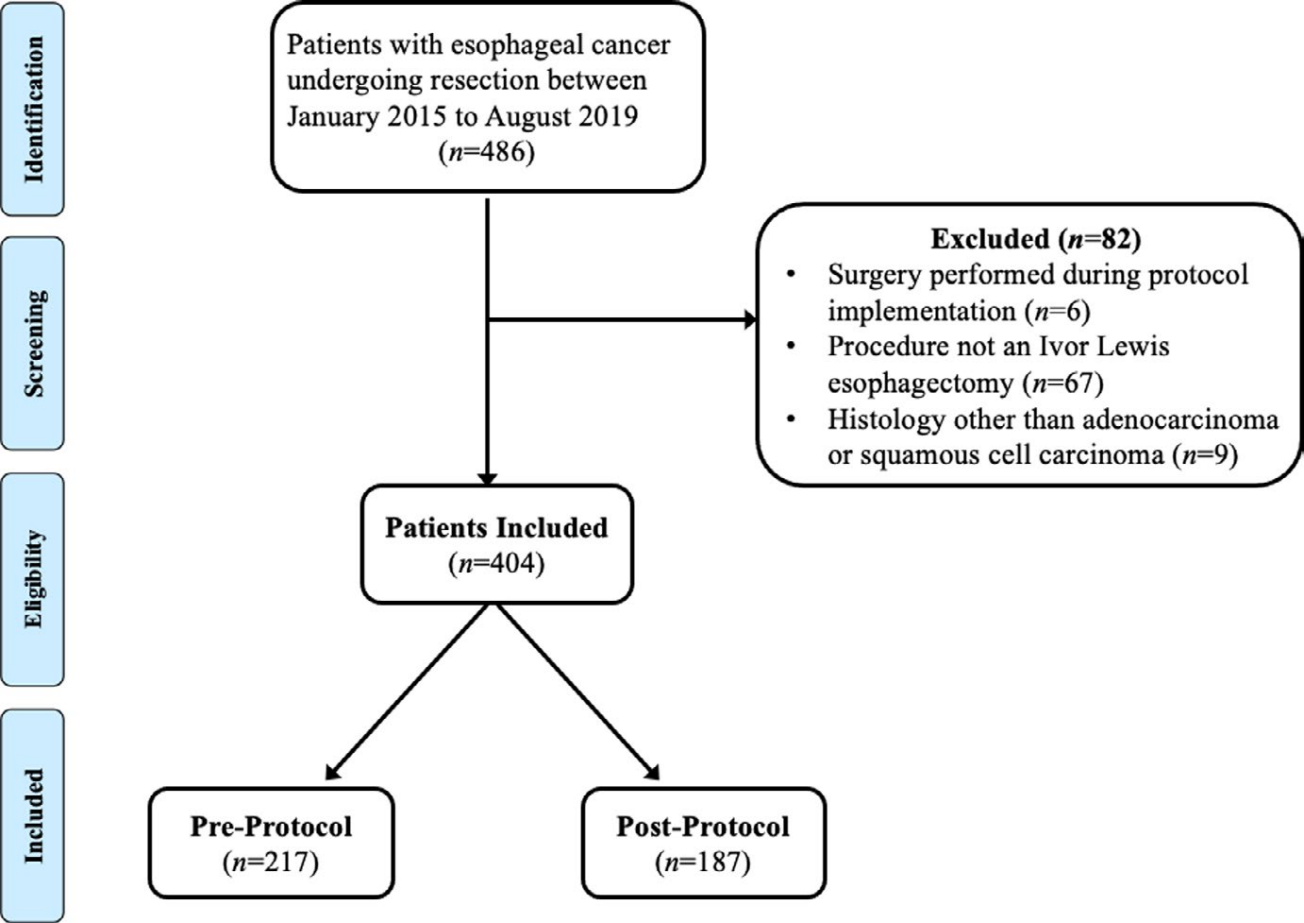
Study flow chart describing the inclusion criteria and exclusion criteria of all included patients

A total of 410 patients with histologically proven EAC or ESCC underwent Ivor Lewis esophagectomy from January 2015 to August 2019. Of these patients, six underwent resection during the month of protocol initiation and were excluded. The remaining 404 patients were included for analysis, with 217 patients in the preprotocol group and 187 patients in the postprotocol group (Figure 3).

All relevant baseline patient and disease characteristics are displayed in Table 1. There were no statistically significant differences in patient demographics, smoking history, comorbidities, or disease characteristics between the two groups. Among both groups, most patients were male (83.7%), white (88.4%), and undergoing resection for EAC (92.8%). Most patients received neoadjuvant treatment prior to surgical resection (82.7%), which most often consisted of concurrent chemoradiotherapy.

**TABLE 1:** Relevant baseline demographic, clinical, and pathologic characteristics of all included patients within the cohort (*n* = 404)

	Protocol (−) (*n* = 217)	Protocol (+) (*n* = 187)	*p*
Sex			0.06
Male	189 (87%)	149 (80%)	
Female	28 (13%)	38 (20%)	
Age, years	64 [52–85]	66 [55–87]	0.16
Race			>0.99
White	192 (88%)	166 (89%)	
Black	3 (1%)	1 (1%)	
Asian	12 (6%)	12 (6%)	
Other	10 (5%)	8 (4%)	
Comorbidity			
Pulmonary	28 (13%)	27 (14%)	0.67
Cardiac	127 (59%)	122 (65%)	0.18
Endocrine	39 (18%)	38 (20%)	0.61
Renal	5 (2%)	5 (3%)	>0.99
Smoking status			>0.99
Never	74 (34%)	63 (34%)	
Former/current	143 (66%)	124 (66%)	
Pretreatment BMI, kg/m^2^			0.96
Underweight (<18.5 kg/m^2^)	3 (1.4%)	2 (1.1%)	
Normal (≥18.5–24.9 kg/m^2^)	46 (21.2%)	43 (23.0%)	
Overweight (≥25.0–29.9 kg/m^2^)	89 (41.0%)	77 (41.2%)	
Obese (≥30 kg/m^2^)	79 (36.4%)	65 (34.8%)	
Tumor type			0.85
Adenocarcinoma	202 (93%)	173 (93%)	
Squamous cell carcinoma	15 (7%)	14 (8%)	
Clinical stage			0.21
I/II	55 (24%)	45 (24%)	
III	124 (57%)	120 (64%)	
IV	38 (18%)	22 (12%)	
Neoadjuvant			0.35
None	43 (20%)	27 (14%)	
Chemotherapy alone	6 (3%)	6 (3%)	
Chemoradiation	168 (77%)	154 (82%)	
ASA classification			0.37
ASA II	33 (15%)	20 (11%)	
ASA III	170 (78%)	152 (81%)	
ASA IV	14 (7%)	15 (8%)	
Preoperative BMI, kg/m^2^			>0.99
Underweight (<18.5 kg/m^2^)	4 (1.8%)	3 (1.6%)	
Normal (≥18.5–24.9 kg/m^2^)	58 (26.7%)	52 (27.8%)	
Overweight (≥25.0–29.9 kg/m^2^)	85 (39.2%)	73 (39.0%)	
Obese (≥30 kg/m^2^)	70 (32.3%)	59 (31.6%)	
Surgical approach			**<0.001**
Open	114 (53%)	51 (27%)	
Minimally invasive	103 (47%)	136 (73%)	
Pyloric drainage			**<0.001**
None	127 (59%)	139 (74%)	
Botox	14 (6%)	7 (4%)	
Pyloroplasty	27 (12%)	34 (18%)	
Pyloromyotomy	49 (23%)	7 (4%)	

Note

Data are no. (%) or median [range]. Statistical tests performed: Fisher's exact test; Wilcoxon rank‐sum test. Bold values indicate statistically significance (*p* < 0.05).

Abbreviations: ASA, American Society of Anesthesiology; BMI, body mass index.

At initial consultation, there were no significant differences in BMI categories or preoperative weight loss between groups. Most patients were overweight (41.1%) or obese (35.6%), with 22.0% of patients having a BMI within the normal range and only 1.2% of patients having a BMI classified as underweight. At this visit, a total of 63 patients (32 patients in the preprotocol group and 31 patients in the postprotocol group) reported an unintentional weight loss >10% of their normal body weight.

At time of surgery, 30 patients (14%) in the preprotocol group and 20 patients (13%) in the postprotocol group had an unintentional weight loss equal to or >10% of their body weight measured at initial consultation. Similarly, based on preoperative BMI, the majority of patients in both groups were considered overweight (39.1%) or obese (31.9%), with 27.2% of patients having a BMI within the normal range and only 1.7% of patients having a BMI classified as underweight.

All patients underwent Ivor Lewis esophagectomy with reconstruction utilizing a gastric conduit and intrathoracic anastomosis. Despite these similarities, significant differences were observed in the approach between groups. Specifically, the proportion of cases utilizing a minimally invasive approach was significantly higher in the postprotocol group (73%) compared with the preprotocol group (48%) (*p* < 0.001). Additionally, cases performed with a pyloric drainage procedure such as Botox injection, pyloroplasty, and pyloromyotomy to improve gastric emptying and minimize potential negative sequelae attributed to delayed gastric emptying was significantly lower in the postprotocol group (41% vs. 26%; *p* < 0.001).

In‐hospital mortality, 30‐day mortality, 90‐day mortality, 30‐day readmission, and prevalence of postoperative complication did not differ significantly between groups (Table 2). Median length of hospital stay was significantly shorter in the postprotocol group (*p* < 0.001). Compared with the preprotocol group, patients in the postprotocol group had a significantly shorter median time in days to diet initiation (*p* < 0.001) as well as a shorter median time in days to feeding tube removal (*p* = 0.012). At outpatient follow‐up, 25 patients (12%) in the preprotocol group demonstrated a postoperative weight loss ≥10% compared with nine patients (5%) in the postprotocol group (*p* = 0.011). Of note, when analysis was restricted to patients receiving neoadjuvant therapy prior to surgery, median days to diet initiation (*p* = 0.001), days to feeding tube removal (*p* = 0.011), length of hospital stay (*p* < 0.001), and weight loss at follow‐up (*p* = 0.005) remained significantly reduced in the post protocol group.

**TABLE 2:** Results of main study outcomes among all included patients within the cohort (*n* = 404)

	Protocol (−) (*n* = 217)	Protocol (+) (*n* = 187)	*p*
Days to diet initiation	8 [7–12]	7 [6–8]	**<0.001**
Days to jejunostomy removal	30 [22–48]	26 [18–38]	**0.012**
Length of hospital stay, days	10 [8–13]	8 [7–11]	**<0.001**
Weight loss at follow‐up visit, kg	3.4 [0.7–6.7]	2.4 [0.8–4.7]	**0.002**
Postoperative complications			
Serious complication	58 (27%)	47 (25%)	0.72
Pulmonary	62 (28%)	52 (27%)	0.91
Cardiac	45 (21%)	31 (17%)	0.31
Gastrointestinal	54 (25%)	48 (26%)	0.91
Anastomotic complication	38 (18%)	29 (16%)	0.69
Chylothorax	6 (3%)	9 (5%)	0.30
Jejunostomy malfunction	12 (6%)	15 (8%)	0.33
Urologic	23 (11%)	19 (10%)	>0.99
Wound infection	12 (6%)	12 (6%)	0.83
Neurologic/psychologic	8 (4%)	9 (5%)	0.63
Hospital mortality	5 (2%)	1 (1%)	0.22
30‐day readmission	35 (16%)	27 (14%)	0.68
30‐day mortality	5 (2%)	1 (1%)	0.22
90‐day mortality	11 (5%)	3 (2%)	0.10

Note

Data are no. (%) or median [interquartile range]. Statistical tests performed: Fisher's exact test; Wilcoxon rank‐sum test. Bold values indicate statistically significance (*p* < 0.05).

To address potential bias caused by differences in operative technique between groups, analysis of postoperative outcomes was then limited to patients who underwent a minimally invasive resection. Similar to previous results, when compared with the preprotocol group, there were significant reductions in median length of hospital stay (*p* < 0.001), time to initiation of diet (*p* < 0.001), time to feeding tube removal (*p* = 0.012), and postoperative weight loss (*p* = 0.002) in the postprotocol group (Table 3). In‐hospital mortality, 30‐day mortality, 90‐day mortality, 30‐day readmission, and incidence of serious complication remained similar between groups. At outpatient follow‐up, 14 patients (14%) in the preprotocol group who underwent minimally invasive resection demonstrated a postoperative weight loss ≥10% compared with four patients (3%) in the postprotocol group (*p* = 0.001).

**TABLE 3:** Results of main study outcomes limited to patients undergoing minimally invasive resection

	Protocol (−) (*n* = 103)	Protocol (+) (*n* = 136)	*p*
Days to diet initiation	8 [6–14]	7 [6–8]	**<0.001**
Days to jejunostomy removal	30 [23–48]	26 [20–42]	**0.019**
Length of hospital stay, days	8 [7–12]	8 [7–9]	**0.02**
Weight loss at follow‐up visit, kg	2.8 [0.4–7.5]	2.1 [−0.2–4.4]	**0.03**
Postoperative complications			
Serious complication	23 (22%)	31 (23%)	>0.99
Pulmonary	25 (24%)	33 (24%)	>0.99
Cardiac	23 (20%)	19 (14%)	0.22
Gastrointestinal	24 (23%)	33 (24%)	0.88
Anastomotic complication	18 (18%)	24 (18%)	>0.99
Chylothorax	4 (4%)	5 (4%)	>0.99
Jejunostomy malfunction	3 (3%)	11 (8%)	0.10
Urologic	10 (10%)	9 (7%)	0.47
Wound infection	2 (2%)	7 (5%)	0.31
Neurologic/psychologic	2 (2%)	4 (3%)	0.70
Hospital mortality	1 (1%)	0 (0%)	0.43
30‐day readmission	19 (18%)	19 (14%)	0.38
30‐day mortality	1 (1%)	0 (0%)	0.43
90‐day mortality	1 (1%)	2 (2%)	>0.99

Note

Data are no. (%) or median [interquartile range]. Statistical tests performed: Fisher's exact test; Wilcoxon rank‐sum test. Bold values indicate statistically significance (*p* < 0.05).

The impact of the nutrition protocol on all continuous outcome variables was then adjusted for age, gender, American Society of Anesthesiologists physical status classification, smoking history, neoadjuvant therapy, histological type of tumor, pretreatment BMI, pyloric drainage procedure, and adjuvant treatment using multiple linear regression modeling. In the postprotocol group, nutrition protocol remained significantly associated with a reduction in median days to diet initiation (*p* = 0.012), days to feeding tube removal (*p* = 0.030), length of hospital stay (*p* = 0.016), and weight loss at follow‐up (*p* = 0.001). Similarly, when analysis was restricted to patients who underwent minimally invasive resection, in the postprotocol group, nutrition protocol remained significantly associated with a decrease in median days to diet initiation (*p* = 0.004) and weight loss at follow‐up (*p* = 0.002), but there was no longer a statistically significant reduction in median length of hospital stay and days to feeding tube removal.

## 4 DISCUSSION

Surgical resection remains the cornerstone of curative treatment of EC. While overall advances in operative technique have significantly improved perioperative outcomes and long‐term survival in these patients, esophagectomy remains one of the most technically challenging and potentially morbid procedures in thoracic surgery, with up to 60% of patients developing postoperative complications.^22^, ^23^, ^24^, ^25^, ^26^, ^27^, ^28^, ^29^, ^30^ As a result, there is great interest in developing methods to reduce perioperative morbidity and mortality.

Unintentional weight loss and malnutrition are important prognostic factors predicting increased risk of postoperative complication and reduced treatment efficacy and survival.^2^, ^3^, ^4^, ^5^, ^6^, ^7^, ^8^, ^9^, ^31^, ^32^ This is particularly relevant in EC considering that preoperative weight loss of >10% has been shown to be associated with reduced overall 5‐year survival following resection,^9^, ^31^, ^32^ which is present in up to 85% of patients with EC at time of diagnosis.^2^, ^3^, ^4^, ^5^, ^6^, ^7^, ^8^, ^9^ Therefore, nutritional support and prevention of malnutrition are imperative in these patients and have been shown to reduce the incidence of life‐threatening complications and shorten postoperative hospital stay.^33^, ^34^

Results of the present study support the addition of intensive nutritional monitoring to standard perioperative protocols. Preoperative assessment of malnutrition provides an opportunity to intervene prior to surgery, improve patient education regarding postoperative nutrition goals, and may reduce postoperative morbidity. Patients in the postprotocol group initiated oral intake faster, had lost less weight at follow‐up, experienced reduced length of hospital stay, and had earlier discontinuation of jejunostomy feeds following discharge. Despite these improvements, a substantial proportion of patients in both groups were affected by unintentional weight loss and malnutrition, suggesting that our nutritional support pathway as well as current perioperative protocols need improvement.^35^

With continuous advances in minimally invasive surgical techniques, it is not surprising that a greater proportion of patients underwent a minimally invasive resection in the postprotocol study group. Several high‐powered randomized controlled trials and metanalyses have reported that a minimally invasive resection provides identical oncological outcomes as open techniques but with reduced intraoperative blood loss, postoperative complication, and length of hospital stay.^28^, ^36^, ^37^, ^38^ Other benefits of minimally invasive resection as compared with open surgery reported in the literature include improved‐health related quality of life, postoperative functional recovery, and overall physical functioning.^39^, ^40^, ^41^

There was also a significant reduction in the number of pyloric drainage procedures performed in the postprotocol group. Typically, pyloric drainage is performed to minimize the negative sequelae attributed to delayed gastric emptying; however, multiple studies have failed to show any significant improvements in postoperative complications in patients undergoing these procedures.^42^, ^43^ Additionally, previous work demonstrated increased long‐term morbidity in patients who received Botulinum injection compared with patients who did not receive any pyloric drainage procedure.^44^

A unique aspect of our program is the continued follow‐up with patients after discharge. Traditionally, perioperative programs conclude once patients are discharged. However, the first 2 weeks following hospital discharge has been shown to represent a period of accelerated weight loss, emphasizing the importance of nutritional support during this period.^45^ Close postoperative follow‐up allows for regular and scheduled symptom monitoring by the outpatient dietitian and may assist in earlier identification and assessment of significant weight loss and malnutrition. More research is needed to evaluate the impact of perioperative nutrition protocols on long‐term survival, patient satisfaction, and patient compliance with nutrition goals.

### 4.1 Limitations

Our study was a retrospective analysis of a single‐center experience; therefore, results may not be generalizable to other populations. Despite our efforts to control for confounding factors with multivariable linear regression modeling as well as repeating all statistical analysis in patients who underwent minimally invasive resection, there is still a risk of selection bias due to the nonrandomized study design. There may have been other factors that could have accounted for weight loss that we did not capture owing to the fact that this is a retrospective study. It would have been ideal to utilize a randomized controlled trial study design. We also acknowledge that, while feedback was obtained through qualitative interviews, more quantitative data are needed to assess patient satisfaction and overall compliance with the program.

## 5 CONCLUSION

In summary, perioperative nutrition support programs may help reduce weight loss and length of stay for EC patients undergoing esophagectomy. Initial results suggest that more aggressive nutritional supplement programs are feasible and may lead to improved postoperative outcomes in patients undergoing esophagectomy. Larger randomized prospective trials are needed to better guide implementation of these protocols, but the initial results are quite promising.

## CONFLICTS OF INTEREST

David R. Jones serves as a consultant for AstraZeneca and on a Clinical Trial Steering Committee for Merck. Daniela Molena serves as a consultant for Johnson & Johnson, Urogen, and Boston Scientific. All other authors have no conflicts.

## ETHICS

All relevant ethical guidelines were followed in the conduct of this study.

## Data Availability

Data are available from the corresponding author on request.

## References

[1] Anandavadivelan P , Lagergren P . Cachexia in patients with oesophageal cancer. Nat Rev Clin Oncol. 2016;13(3):185‐198. 26573424 10.1038/nrclinonc.2015.200

[2] Bozzetti F . Screening the nutritional status in oncology: a preliminary report on 1,000 outpatients. Support Care Cancer. 2009;17(3):279‐284. 18581148 10.1007/s00520-008-0476-3

[3] Mariette C , De Botton M‐L , Piessen G . Surgery in esophageal and gastric cancer patients: what is the role for nutrition support in your daily practice? Ann Surg Oncol. 2012;19(7):2128‐2134. 22322948 10.1245/s10434-012-2225-6

[4] Hébuterne X , Lemarié E , Michallet M , de Montreuil CB , Schneider SM , Goldwasser F . Prevalence of malnutrition and current use of nutrition support in patients with cancer. JPEN J Parenter Enteral Nutr. 2014;38(2):196‐204. 24748626 10.1177/0148607113502674

[5] Bosaeus I , Daneryd P , Lundholm K . Dietary intake, resting energy expenditure, weight loss and survival in cancer patients. J Nutr. 2002;132(11 suppl):3465s‐3466s. 12421871 10.1093/jn/132.11.3465S

[6] Caccialanza R , Goldwasser F , Marschal O , et al. Unmet needs in clinical nutrition in oncology: a multinational analysis of real‐world evidence. Ther Adv Med Oncol. 2020;12:1758835919899852. 32110247 10.1177/1758835919899852 PMC7025419

[7] Baracos VE . Cancer‐associated malnutrition. Eur J Clin Nutr. 2018;72(9):1255‐1259. 30185853 10.1038/s41430-018-0245-4

[8] Grace EM , Shaw C , Lalji A , Mohammed K , Andreyev HJN , Whelan K . Nutritional status, the development and persistence of malnutrition and dietary intake in oesophago‐gastric cancer: a longitudinal cohort study. J Hum Nutr Diet. 2018;31(6):785‐792. 30033545 10.1111/jhn.12588

[9] Riccardi D , Allen K . Nutritional management of patients with esophageal and esophagogastric junction cancer. Cancer Control. 1999;6(1):64‐72. 10758536 10.1177/107327489900600106

[10] Awad S , Tan BH , Cui H , et al. Marked changes in body composition following neoadjuvant chemotherapy for oesophagogastric cancer. Clin Nutr. 2012;31(1):74‐77. 21875767 10.1016/j.clnu.2011.08.008

[11] Miller KR , Bozeman MC . Nutrition therapy issues in esophageal cancer. Curr Gastroenterol Rep. 2012;14(4):356‐366. 22730015 10.1007/s11894-012-0272-6

[12] Deans DAC , Tan BH , Wigmore SJ , et al. The influence of systemic inflammation, dietary intake and stage of disease on rate of weight loss in patients with gastro‐oesophageal cancer. Br J Cancer. 2009;100(1):63‐69. 19127266 10.1038/sj.bjc.6604828 PMC2634686

[13] Baker M , Halliday V , Williams RN , Bowrey DJ . A systematic review of the nutritional consequences of esophagectomy. Clin Nutr. 2016;35(5):987‐994. 26411750 10.1016/j.clnu.2015.08.010 PMC5410167

[14] Yamasaki M , Miyata H , Yasuda T , et al. Impact of the route of reconstruction on post‐operative morbidity and malnutrition after esophagectomy: a multicenter cohort study. World J Surg. 2015;39(2):433‐440. 25315089 10.1007/s00268-014-2819-1

[15] Steenhagen E , van Vulpen JK , van Hillegersberg R , May AM , Siersema PD . Nutrition in peri‐operative esophageal cancer management. Expert Rev Gastroenterol Hepatol. 2017;11(7):663‐672. 28454509 10.1080/17474124.2017.1325320

[16] Ligthart‐Melis GC , Weijs PJM , te Boveldt ND , et al. Dietician‐delivered intensive nutritional support is associated with a decrease in severe postoperative complications after surgery in patients with esophageal cancer. Dis Esophagus. 2013;26(6):587‐593. 23237356 10.1111/dote.12008

[17] Huddy JR , Huddy FMS , Markar SR , Tucker O . Nutritional optimization during neoadjuvant therapy prior to surgical resection of esophageal cancer‐a narrative review. Dis Esophagus. 2018;31(1):1‐11. 10.1093/dote/dox110 29024949

[18] Amin MB , Edge SB . AJCC Cancer Staging Manual. 8th ed. Springer; 2017.

[19] Clinical guidelines on the identification, evaluation, and treatment of overweight and obesity in adults‐‐the evidence report. National Institutes of Health. Obes Res. 1998;6(suppl 2):51s‐209s. 9813653

[20] Obesity: preventing and managing the global epidemic. Report of a WHO consultation. World Health Organ Tech Rep Ser. 2000;894:i‐xii, 1–253. 11234459

[21] Clavien PA , Barkun J , de Oliveira ML , et al. The Clavien‐Dindo classification of surgical complications: five‐year experience. Ann Surg. 2009;250(2):187‐196. 19638912 10.1097/SLA.0b013e3181b13ca2

[22] Ahmadi N , Crnic A , Seely AJ , et al. Impact of surgical approach on perioperative and long‐term outcomes following esophagectomy for esophageal cancer. Surg Endosc. 2018;32(4):1892‐1900. 29067584 10.1007/s00464-017-5881-6

[23] Gottlieb‐Vedi E , Kauppila JH , Malietzis G , Nilsson M , Markar SR , Lagergren J . Long‐term survival in esophageal cancer after minimally invasive compared to open esophagectomy: a systematic review and meta‐analysis. Ann Surg. 2019;270(6):1005‐1017. 30817355 10.1097/SLA.0000000000003252

[24] Booka E , Takeuchi H , Kikuchi H , et al. Recent advances in thoracoscopic esophagectomy for esophageal cancer. Asian J Endosc Surg. 2019;12(1):19‐29. 30590876 10.1111/ases.12681

[25] Lv L , Hu W , Ren Y , Wei X . Minimally invasive esophagectomy versus open esophagectomy for esophageal cancer: a meta‐analysis. Onco Targets Ther. 2016;9:6751‐6762. 27826201 10.2147/OTT.S112105 PMC5096744

[26] Sihag S , Kosinski AS , Gaissert HA , Wright CD , Schipper PH . Minimally invasive versus open esophagectomy for esophageal cancer: a comparison of early surgical outcomes from the Society of Thoracic Surgeons National Database. Ann Thorac Surg. 2016;101(4):1281‐1288; discussion 1288–1289. 26704412 10.1016/j.athoracsur.2015.09.095

[27] Taurchini M , Cuttitta A . Minimally invasive and robotic esophagectomy: state of the art. J vis Surg. 2017;3:125. 29078685 10.21037/jovs.2017.08.23 PMC5639027

[28] Straatman J , van der Wielen N , Cuesta MA , et al. Minimally invasive versus open esophageal resection: three‐year follow‐up of the previously reported randomized controlled trial: the TIME Trial. Ann Surg. 2017;266(2):232‐236. 28187044 10.1097/SLA.0000000000002171

[29] Atkins BZane , Shah AS , Hutcheson KA , et al. Reducing hospital morbidity and mortality following esophagectomy. Ann Thorac Surg. 2004;78(4):1170‐1176; discussion 1170–1176. 15464465 10.1016/j.athoracsur.2004.02.034

[30] Zingg U , Smithers BM , Gotley DC , et al. Factors associated with postoperative pulmonary morbidity after esophagectomy for cancer. Ann Surg Oncol. 2011;18(5):1460‐1468. 21184193 10.1245/s10434-010-1474-5

[31] van der Schaaf MK , Tilanus HW , van Lanschot JJB , et al. The influence of preoperative weight loss on the postoperative course after esophageal cancer resection. J Thorac Cardiovasc Surg. 2014;147(1):490‐495. 24060365 10.1016/j.jtcvs.2013.07.072

[32] Hynes O , Anandavadivelan P , Gossage J , Johar AM , Lagergren J , Lagergren P . The impact of pre‐ and post‐operative weight loss and body mass index on prognosis in patients with oesophageal cancer. Eur J Surg Oncol. 2017;43(8):1559‐1565. 28655483 10.1016/j.ejso.2017.05.023

[33] Fujita T , Daiko H , Nishimura M . Early enteral nutrition reduces the rate of life‐threatening complications after thoracic esophagectomy in patients with esophageal cancer. Eur Surg Res. 2012;48(2):79‐84. 22377820 10.1159/000336574

[34] Mazaki T , Ebisawa K . Enteral versus parenteral nutrition after gastrointestinal surgery: a systematic review and meta‐analysis of randomized controlled trials in the English literature. J Gastrointest Surg. 2008;12(4):739‐755. 17939012 10.1007/s11605-007-0362-1

[35] Benton K , Thomson I , Isenring E , Mark Smithers B , Agarwal E . An investigation into the nutritional status of patients receiving an Enhanced Recovery After Surgery (ERAS) protocol versus standard care following Oesophagectomy. Supp Care Cancer. 2018;26(6):2057‐2062. 10.1007/s00520-017-4038-4 29368029

[36] Yibulayin W , Abulizi S , Lv H , Sun W . Minimally invasive oesophagectomy versus open esophagectomy for resectable esophageal cancer: a meta‐analysis. World J Surg Oncol. 2016;14(1):304. 27927246 10.1186/s12957-016-1062-7 PMC5143462

[37] Zhou C , Zhang LI , Wang H , et al. Superiority of minimally invasive oesophagectomy in reducing in‐hospital mortality of patients with resectable oesophageal cancer: a meta‐analysis. PLoS One. 2015;10(7):e0132889. 26196135 10.1371/journal.pone.0132889 PMC4509855

[38] Nagpal K , Ahmed K , Vats A , et al. Is minimally invasive surgery beneficial in the management of esophageal cancer? A meta‐analysis. Surg Endosc. 2010;24(7):1621‐1629. 20108155 10.1007/s00464-009-0822-7

[39] Kauppila JH , Xie S , Johar A , Markar SR , Lagergren P . Meta‐analysis of health‐related quality of life after minimally invasive versus open oesophagectomy for oesophageal cancer. Br J Surg. 2017;104(9):1131‐1140. 28632926 10.1002/bjs.10577

[40] van der Sluis PC , van der Horst S , May AM , et al. Robot‐assisted minimally invasive thoracolaparoscopic esophagectomy versus open transthoracic esophagectomy for resectable esophageal cancer: a randomized controlled trial. Ann Surg. 2019;269(4):621‐630. 30308612 10.1097/SLA.0000000000003031

[41] Maas KW , Cuesta MA , van Berge Henegouwen MI , et al. Quality of life and late complications after minimally invasive compared to open esophagectomy: results of a randomized trial. World J Surg. 2015;39(8):1986‐1993. 26037024 10.1007/s00268-015-3100-y PMC4496501

[42] Arya S , Markar SR , Karthikesalingam A , Hanna GB . The impact of pyloric drainage on clinical outcome following esophagectomy: a systematic review. Dis Esophagus. 2015;28(4):326‐335. 24612489 10.1111/dote.12191

[43] Palmes D , Weilinghoff M , Colombo‐Benkmann M , Senninger N , Bruewer M . Effect of pyloric drainage procedures on gastric passage and bile reflux after esophagectomy with gastric conduit reconstruction. Langenbecks Arch Surg. 2007;392(2):135‐141. 17216285 10.1007/s00423-006-0119-4

[44] Nobel T , Tan KS , Barbetta A , et al. Does pyloric drainage have a role in the era of minimally invasive esophagectomy? Surg Endosc. 2019;33(10):3218‐3227. 30535543 10.1007/s00464-018-06607-8 PMC6557699

[45] Wang P , Li Y , Sun H , et al. Analysis of the associated factors for severe weight loss after minimally invasive McKeown esophagectomy. Thorac Cancer. 2019;10(2):209‐218. 30578600 10.1111/1759-7714.12934 PMC6360231

